# Does Frailty influence Inhospital Management and Outcomes of COVID-19 in Older Adults in the US?

**DOI:** 10.1093/geroni/igab046.3477

**Published:** 2021-12-17

**Authors:** Soko Setoguchi, Richard Kennedy, Nathaniel Kuhrt, Timothy Bergquist, Jessica Islam, Heidi Spratt, Vithal Madhira, Joseph Siu

**Affiliations:** 1 Rutgers Robert Wood Johnson Medical School, New Brunswick, New Jersey, United States; 2 University of Alabama at Birmingham, Birmingham, Alabama, United States; 3 New Jersey Medical School, Newark, New Jersey, United States; 4 Sage Bionetworks, Kent, Washington, United States; 5 H. Lee Moffitt Cancer Center and Research Institute, Tampa, Florida, United States; 6 University of Texas Medical Branch, Galveston, Texas, United States; 7 Palila Software LLC, Palila Software LLC, Nevada, United States; 8 University of Nebraska Medical Center, Omaha, Nebraska, United States

## Abstract

Older age has been consistently associated with adverse COVID-19 outcomes. Frailty, a syndrome characterized by declining function across multiple body systems is common in older adults and may increase vulnerability to adverse outcomes among COVID-19 patients. However, the impacts of frailty on COVID-19 management, severity, or outcomes have not been well characterized in a large, representative US population. Using the National COVID Cohort Collaborative, a multi-institutional US repository for COVID-19 research, we calculated the Hospital Frailty Risk Score (HFRS), a validated EHR-based frailty score, among COVID-19 inpatients age ≥ 65. We examined patient demographics and comorbidities, length of stay (LOS), systemic corticosteroid and remdesivir use, ICU admission, and inpatient mortality across subgroups by HFRS score. Among 58,964 inpatients from 53 institutions (51% male, 65% White, 18% Black, 9% Hispanic, mean age 75, mean Charlson comorbidity count 3.0, and median LOS 7 days), 38,692 (66%), 4,180 (7%), 3,531 (6%), 3,525 (6%) and 7,862 (13%) had HFRS scores of 0-1, 2, 3, 4, and >=5 , respectively. Frailty was only moderately correlated with age and comorbidity (□=0.178 and 0.348, respectively, p<0.001). Overall, 34% received systemic corticosteroid and 19% received remdesivir. We observed 4% ICU admissions and 16% inpatient death. Among non-ICU admissions, after adjusting for demographics and comorbidities, frailty (HFRS ≥ 2) was associated with 79% greater systemic corticosteroid use and 22% greater remdesivir use, whereas a higher HRFS score was marginally associated with higher rates of severe COVID disease, inpatient death, or ICU admission.

